# Identification of active metabolites from *Gentiana scabra* Bunge ethanol-water extract for chicken metatarsal fracture repair

**DOI:** 10.3389/fphar.2025.1706754

**Published:** 2025-11-25

**Authors:** Yi Jiang Liu, Yue Min Wang, Bin Jiang, Hou Qiang Luo, Su Zhen Liu, Shuai Luo

**Affiliations:** 1 College of Animal Sciences, Wenzhou Vocational College of Science Technology, Wenzhou, China; 2 Zhejiang Provincial Key Laboratory of Biometrology and Inspection and Quarantine, College of Life Sciences, China Jiliang University, Hangzhou, China

**Keywords:** fracture healing, active metabolites, *Gentiana scabra* Bunge, column chromatography, HPLC, LC-MS, X-ray

## Abstract

**Introduction:**

Fractures are common clinical conditions. *Gentiana scabra* Bunge extract (GSE) exhibits multiple biological activities, including anti-nociceptive and anti-inflammatory effects. However, its therapeutic effects and active metabolites regarding fracture healing remain poorly understood. In this study, we aimed to investigate the role and active metabolites of GSE in fracture healing by using chicken fracture model.

**Methods:**

Active metabolites in GSE were identified and isolated via column chromatography, HPLC, and LC-MS. Fracture repair was assessed by X-ray, and the outcomes were quantified with ImageJ software.

**Results:**

The results demonstrated that GSE mother liquor and its fractions GSE30, GSE50, GSE70, GSE90 promoted fracture healing. Compared to the control group, the callus area in the GSE group was increased by 1.8-fold on day 3 and by 2.0-fold on day 10. The most favorable healing effect was observed in the GSE50 group, this fraction was primarily composed of gentisin (27.6%), gentiopicroside (7.3%) and isoorientin (65.1%). A notable dose-response relationship was observed between gentisin and isoorientin concentrations in GSE and bone repair outcomes.

**Discussion:**

Our findings demonstrate that the active metabolites in GSE are potent agents for accelerating fracture repair. This suggests that gentisin, gentiopicroside and isoorientin is a promising candidate for further preclinical development, and its mechanism of action warrants deeper investigation.

## 1 Introduction

Fracture repair has become a hotspot in medical research due to its high incidence rate and prolonged recovery period (Dong et al., 2022). Traditional Chinese medicine (TCM) applications have demonstrated the ability to accelerate fracture recovery in animal models, as evidenced by clinical trial findings (Ding et al., 2023; Ye et al., 2023; Zhang et al., 2023). However, the complexity of TCM metabolitess and the specificity of botanical drugs formulations limit the development of standardized drugs for fracture healing. Therefore, expanding the therapeutic applications of TCM in fracture treatment while identifying the effective bioactive metabolites and elucidating their potential mechanisms of action has become a key research focus in the field of bone injury.

The application of traditional Chinese medicine (TCM) in fracture treatment often originates from its observed pharmacological effects, with its potential mechanisms of action being elucidated through an integrated analysis of the fracture healing process. Gentiana scabra is a medicinal plant from the Gentianaceae family, primarily utilizing its dried roots and rhizomes (Qu et al., 2015). As a traditional Sichuan botanical medicinal with over 2000 years of application history, it has demonstrated anti-inflammatory, hepatoprotective, and hypolipidemic effects (Zheng et al., 2022; Yang et al., 2019; Wang et al., 2023a). For instance, Gentiana scabra extracts (GSE) have shown therapeutic potential for various inflammatory diseases, including contact dermatitis and osteoarthritis (Chen and Wang, 2017; Kim et al., 2019). Additionally, Gentiana scabra exhibits anti-atherosclerotic properties (Lin et al., 2016). Moreover, gentianine, one of its main active metabolites, possesses anti-diabetic effects (Vaidya et al., 2013). Studies demonstrate that gentiopicroside can inhibit RANKL-induced osteoclast formation, suppress the expression of osteoclast-related proteins, and block the activation of NF- κB and JNK signaling pathways, thereby inhibiting osteoclast differentiation and bone resorption (Chen et al., 2018). However, although GSE has attracted widespread attention and been clinically applied (Wang et al., 2021), research on its pharmacodynamics has primarily focused on the hepatoprotective effects of its monomeric compound, gentiopicroside (Dai et al., 2018; Wang et al., 2023b). Notably, studies exploring the effects and mechanisms of GSE on fracture healing remain scarce. Furthermore, systematic screening of GSE’s main active metabolites has yet to be comprehensively conducted.

Studying the functions and active metabolites of Gentiana scabra has provided a novel approach to the application of ethnic medicine. High-performance liquid chromatography (HPLC) coupled with UV and electrospray ionization mass spectrometry (ESI-MS) has played a vital role in identifying active metabolites in Gentiana scabra (Zhang et al., 2020). In addition, GSE has reportedly shown a wide safety margin with no apparent side effects (Stefana et al., 2020). Therefore, active metabolites in GSE may have broad application prospects in fracture healing.

In this study, we aimed to identify the active metabolites in GSE that promote fracture healing. Based on X-ray analysis and J-image quantification, we detected the optimal fraction suitable for fracture repair. Then, LC-MS was employed to identify the chemical metabolites in the optimal fraction, while HPLC was utilized to quantify their contents. Through this approach, we preliminarily explored the pharmacodynamic basis of *Gentiana scabra* active metabolites in fracture healing, clarified the potential active metabolites of *Gentiana scabra* in fracture healing, and provided a theoretical foundation for future research and drug development using *Gentiana scabra* for fracture healing.

## 2 Materials and methods

### 2.1 Preparation and extraction of plant material

#### 2.1.1 Botanical material identification and preparation

The botanical medicine used in this study was ‘Longdan Cao’. Its source plant was identified as *Gentiana scabra* Bunge, belonging to the Gentianaceae family. The material consisted of dried roots and rhizomes, purchased from Beijing Tongrentang, China. The species name was verified using both Kew’s Medicinal Plant Names Services (MPNS) and Plants of the World Online. All extracts were prepared from this authenticated material.

#### 2.1.2 Preparation of *Gentiana scabra* Bunge alcohol-water extract and fractions

The plant material (40 g) was powdered and then extracted twice with 400 mL of 70% ethanol under reflux conditions. After filtration through 3 MM Whatman filter paper (thickness: 0.34 mm). Then divide the liquid into two equal volumes, with one part being stored at 4 °C as the mother liquor (ML), the other extract parts was concentrated to 40 mL by reduced-pressure distillation (Yamato, Japan). The ethanol extract was subsequently subjected to D101 macroporous resin chromatography, where it was first washed with water and then sequentially eluted with 200 mL each of 30%, 50%, 70%, and 90% ethanol to obtain four fractions. Each fraction was concentrated to dryness under reduced pressure and then redissolved in 20 mL of 50% ethanol solution. The yields of the four fractions (GSE30, GSE50, GSE70, and GSE90) were calculated based on the weight of the dried crude drug (w/w).

### 2.2 Chemical profiling of extracts

#### 2.2.1 HPLC analysis

GSE and its fractions (1.0 g) were dissolved in 5 mL of 50% (v/v) ethanol and filtered through 0.45-μm membranes. Quantitative analysis of gentiopicroside, gentisin and isoorientin in GSE and its fractions was conducted using a Waters 600E HPLC system equipped with a Diamond C18 column (250 mm × 4.6 mm, 5 μm particle size) and a Waters 2996 PDA detector. The mobile phase consisted of H_2_O:CH_3_CN (75:25, v/v) with isocratic elution at a flow rate of 1.0 mL/min. The injection volume was 10 μL with column temperature maintained at 30 °C. In brief, Gentiopicroside was detected at 274 nm using the established HPLC method. The chromatogram showed a sharp and symmetric peak for the gentiopicroside standard with a retention time of approximately 2.08 min. Isoorientin was chosen at 340 nm and retention time was 2.11 min. Gentisin was selected at 274 nm and retention time was 3.44 min.

#### 2.2.2 LC-MS analysis

GSE and its fractions were analyzed using both positive and negative electrospray ionization (ESI) modes; however, compound identification was exclusively conducted in negative ionization mode due to enhanced detection sensitivity with improved signal intensity and compound coverage. Full-scan mass spectra were acquired across m/z 200-2000 in both MS and MS/MS modes. Operational parameters included: nitrogen as nebulizing (45 psi), drying (12 L/min), and collision gas; capillary temperature maintained at 300 °C; voltage settings of capillary (3500 V), fragmentor (175 V), skimmer (65 V), and octapole (750 V). Multistage collision energies (15–40 eV in 5 eV increments) were applied for tandem MS analyses. Molecular characterization was performed using MassHunter Workstation (vB.08.00) for accurate mass determination and phytochemical profiling.

#### 2.2.3 HPLC-ESI-Q-MS condition

Chromatographic separation was achieved using an Agilent 1,290 Infinity II UHPLC system hyphenated with an Agilent 6315 quadrupole mass spectrometer equipped with electrospray ionization (ESI) source.

### 2.3 *In vivo* fracture healing assay

#### 2.3.1 Experimental animals and study design

The study was approved by the Ethics Committee of China Jiliang University (Approval No. [2024]032). All procedures were strictly performed in accordance with the institutional animal welfare guidelines. We also acknowledge that a limitation of the experimental design was the absence of prior *in vitro* studies to better optimize the animal model. We hereby solemnly declare that future related research will strictly adhere to a stepwise strategy, mandating the use of *in vitro* models as a compulsory preliminary step to more comprehensively implement the 3R principles. The experiment utilized 18 healthy 8-week-old female Yandang Partridge chickens. At 2 months of age, the chickens were randomly allocated into five experimental groups and one control group. They were housed in separate isolators under controlled environmental conditions (22 °C; 50%–60% relative humidity; 10 lux light intensity) and provided with commercial grower feed (Underwater Killer, China) and water *ad libitum* during the 11-day post-operative observation period. Zoletil® 50 (Virbac, France) was dissolved in physiological saline for anesthesia induction.

#### 2.3.2 Establishment of the chicken metatarsal fracture model

The metatarsal fracture model was established following previously described methods with minor modifications (Hiltunen et al., 1993; Bonnarens and Einhorn, 1984). All chicken were deeply anesthetized using the same dosage of (Zoletil® 50, 0.18 mg/kg) to ensure the absence of muscle tension and movement during the procedure. Chicken were secured on a custom-designed surgical platform, with the right tibia exposed and positioned in a specific support or groove. The fracture site was clearly identified and marked on the skin surface to ensure the impact location was identical for each procedure. A custom-designed, weight-controlled drop impact device was utilized. This apparatus consists of a graduated guide rail and a 3 kg mass. The weight was dropped freely from a fixed height (50 cm), generating an impact energy of 14.7 J (Calculation: Energy = mass × gravitational acceleration × height). This energy level was determined through preliminary experiments to be sufficient for creating a closed transverse fracture without causing excessive soft tissue damage. The 5 mm striker tip at the end of the weight was precisely aligned with the pre-marked mid-shaft of the bone. The target bone was steadily supported at both ends, with a fixed distance of 20 mm between the support points, simulating standard three-point bending mechanics. Successful fracture induction was preliminarily confirmed by the palpable crepitus and abnormal mobility of the limb. Then, X-ray imaging was performed immediately post-procedure for all animals. We established clear inclusion criteria: only animals exhibiting a definitive, completely displaced mid-shaft transverse tibial fracture were included in the subsequent study. Any animal not meeting this standard (excessive comminution or incorrect fracture location) was excluded. The fracture site was then stabilized with 3-6 layers of gauze as a compression pad and three bamboo splints for external fixation. Postoperatively, chickens were transferred to clean cages for recovery. Daily wound monitoring was performed, and fracture repair progression was assessed radiographically at predetermined time points. Fracture wound area and repair area were quantified using J-image software. The fracture healing potential of each fraction was evaluated by calculating the proportion of fracture healing (PFH) (Li et al., 2023), where: PFH = (Fracture wound area on day X - Fracture wound area on day 1)/Fracture wound area on day 1.

#### 2.3.3 Local Administration of extracts and fractions

The active metabolites used in this study were the GSE stock solution and its various fractions. The final concentration of *Gentiana scabra* Bunge in the GSE stock solution was 50 mg/mL. A volume of 1 mL was injected locally per fracture site, resulting in a total administered dose of 50 mg per site. The fraction solutions were obtained by subjecting an equal volume of the GSE stock solution to column chromatography, followed by sequential elution with equal volumes of 30%, 50%, and 70% ethanol. The selection of this concentration was based on previous literature reports (Chinese Pharmacopoeia Commission, 2020). Prior to the formal experiments, we conducted a dose-gradient pilot study (500, 1,000, 1,500 µL). The results indicated that 1,000 µL effectively saturated the gauze while minimizing leakage of the applied liquid, and was therefore selected as the dose for the formal experiments.

A multiple dosing regimen was employed. The administration time points were set at postoperative days 1, 3, 5, 7, and 9 to cover the early stages of fracture healing. This frequency was determined based on preliminary pilot experiments and the timeline of early molecular events in fracture healing, ensuring the maintenance of effective drug concentrations during the most active phase of the cytokine network.

### 2.4 Assessment of fracture repair

#### 2.4.1 X-ray evaluation

At 2 months of age, whole-body radiographs of chickens in each experimental group were obtained using X-ray equipment (Philips, Germany) at 5 kV for 6.0 s. Radiographic examinations of the right tibia and metatarsals were performed on postoperative days 0 (day of surgery), 3, and 10 using the same parameters. Three blinded independent evaluators qualitatively assessed fracture healing status.

### 2.5 Statistical analysis

Experimental data are presented as mean ± SD (n = 3). Statistical comparisons were performed using Student’s t-test for pairwise comparisons and one-way ANOVA with Tukey’s *post hoc* test for multi-group analyses. Statistical significance was defined as P < 0.05 using two-tailed testing.

## 3 Results

### 3.1 Property analysis of GSE fractions obtained by column chromatograph

**FIGURE 1:**
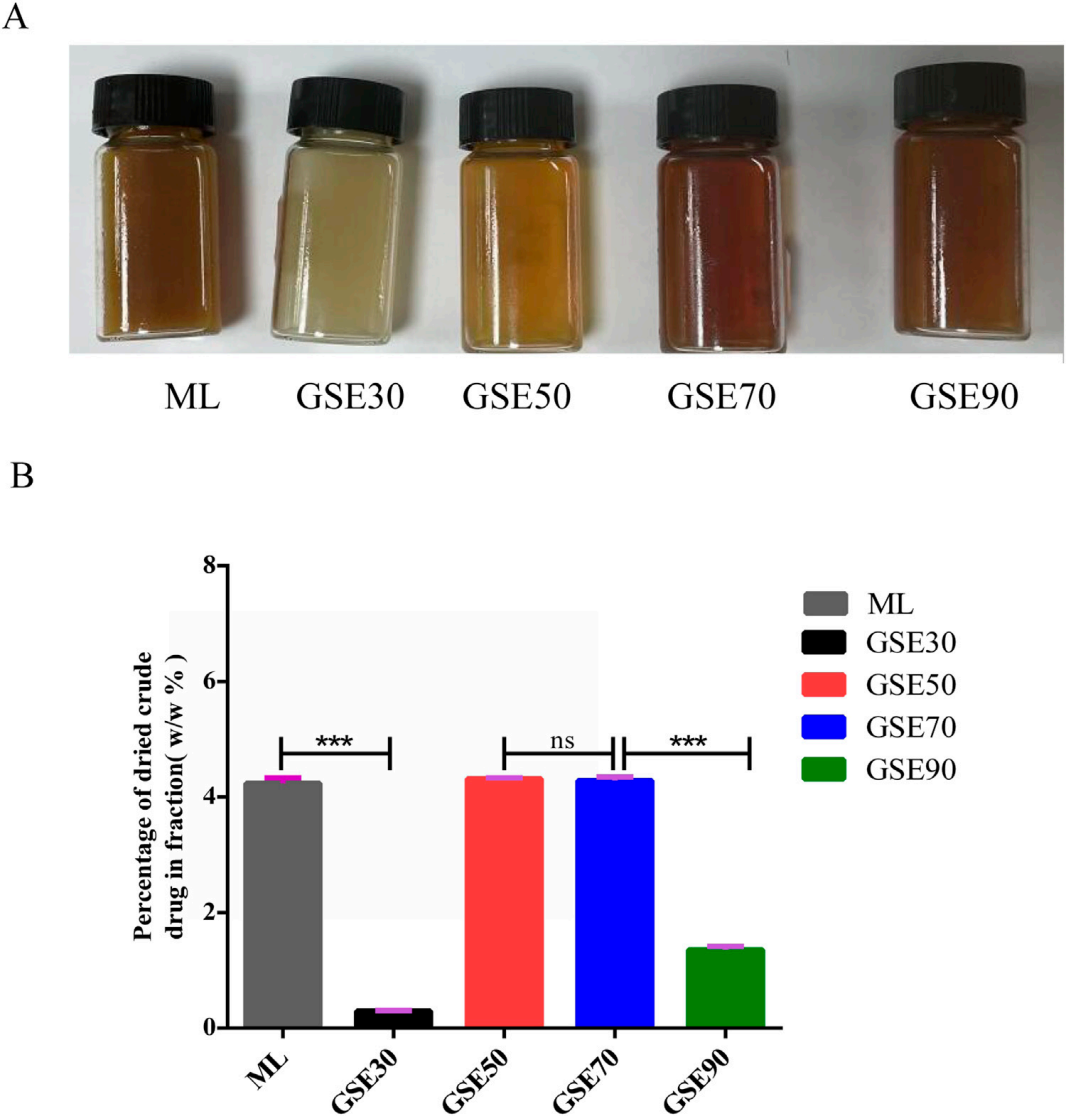
Appearance **(A)** and percentage of dried crude drug. **(B)** GSE mother liquid and its various fractions. The experimental data are expressed using the mean ± standard deviation. n = 3. ns, not significant, ***P < 0.001.

The chromatographic separation protocol was performed as previously described. Fraction differentiation was visually confirmed through distinct chromatic profiles (Figure 1A). Quantitative analysis revealed percentage yields of the dried crude extracts as follows: mother liquor (ML) 4.23%, GSE30 0.30%, GSE50 4.31%, GSE70 4.28%, and GSE90 1.36% (Figure 1B). For experimental standardization, all fractions were concentrated under reduced pressure to viscous residues and reconstituted in 50% (v/v) aqueous ethanol at equivalent concentrations.

### 3.2 Accelerated fracture healing by GSE fractions in avian models

**FIGURE 2:**
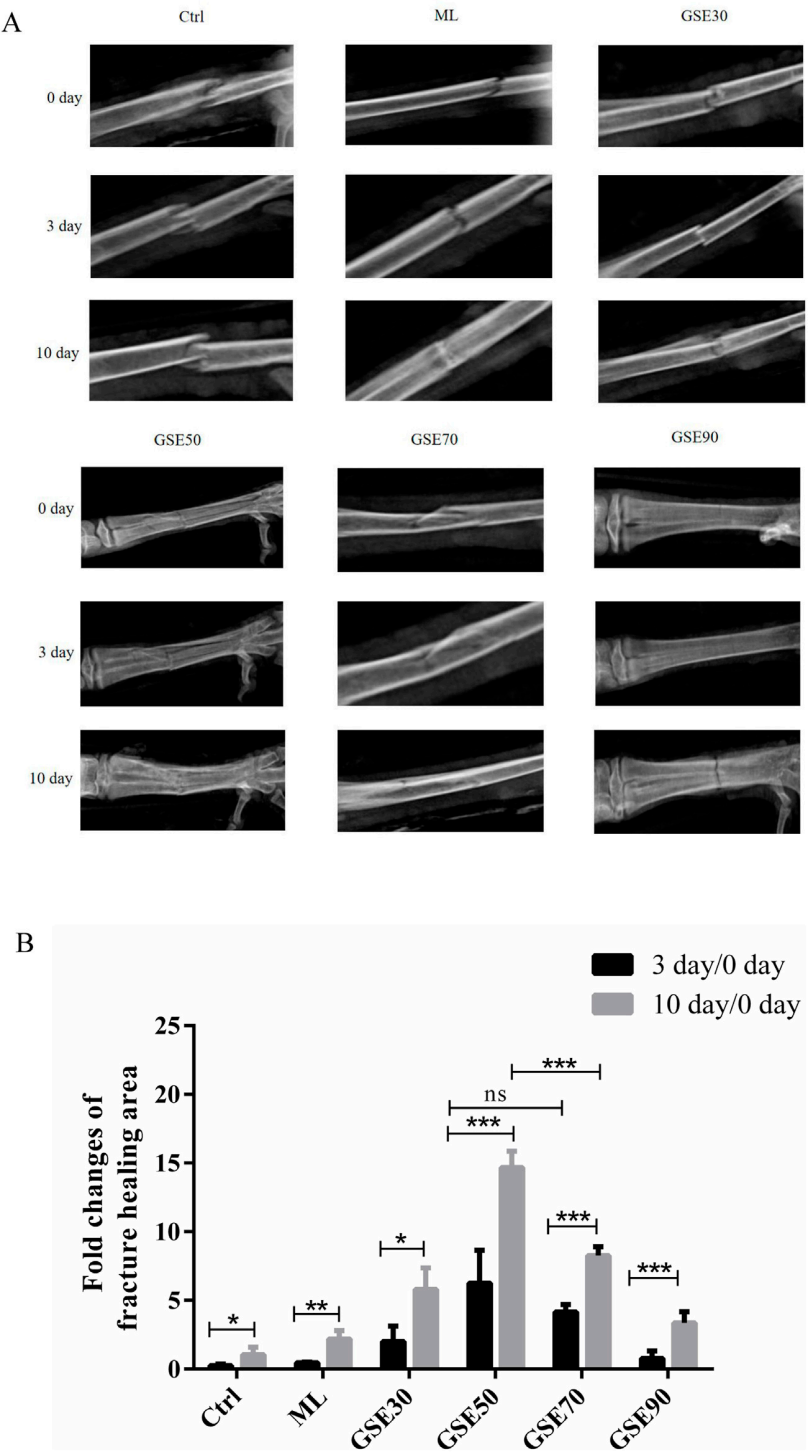
GSE mother liquid and its various fraction accelerate metatarsal bone fracture healing in chicken. **(A)** X-ray evaluate different groups fracture bone repair at different times. **(B)** Each group quantify by using J-image. The experimental data are expressed using the mean ± standard deviation. n = 3. ns, not significant, *p < 0.05, **p < 0.01, ***p < 0.001.

To evaluate the osteogenic potential of GSE and its chromatographic fractions, 18 chickens with standardized metatarsal fractures were randomly allocated into six treatment groups: Control, ML, GSE30, GSE50, GSE70, and GSE90. Topical administration of 1 mL corresponding fraction solutions was performed at fracture sites every 48 h. Radiographic monitoring (Figure 2A) and quantitative morphometric analysis (Figure 2B) revealed differential healing patterns. By postoperative day 3, all intervention groups exhibited enhanced callus formation compared to controls, with GSE50 demonstrating superior osteoregenerative capacity. Microstructural analysis showed initial bony bridging in GSE50-treated fractures. At day 10 post-operation, distinct healing stages were observed: control group maintained clear fracture lines, while GSE30/50/70 groups displayed progressive blurring of fracture margins. The GSE50 cohort exhibited significantly greater callus deposition (p < 0.05 vs. other fractions) with complete osseous bridging, contrasting with partial union in GSE30/70 groups.

### 3.3 Phytochemical profiling of osteogenic fraction GSE50

**FIGURE 3:**
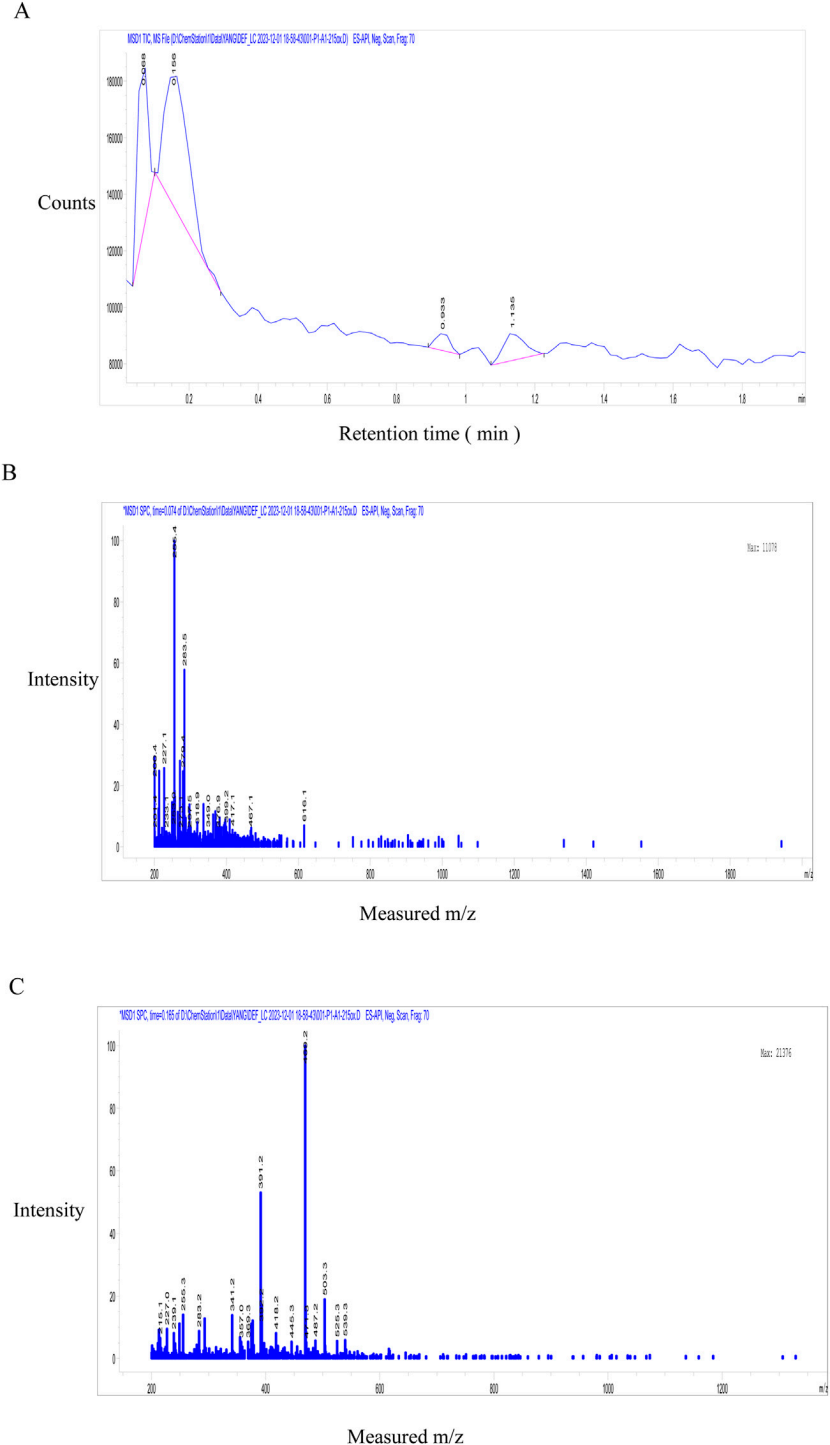
Fractions of GSE50 active component was analyzed by LC-MS. **(A)** HPLC identify peak time for active component in GSE50 fraction. **(B)** MS analysis the composition of GSE50 with peak time of 0.068 min in HPLC. **(C)** MS analysis the composition of GSE50 with peak time of 0.156 min in HPLC.

The investigation focused on GSE50, demonstrating optimal fracture healing efficacy. High-resolution LC-MS analysis (Figure 3) identified six principal phytometabolites: gentisin (m/z 283.5), gentiana alkaloid (m/z 200.4), gentiana trisaccharide (m/z 525.3), gentiopicroside (m/z 356.3), α-amyrin (m/z 457.1), daucosterol (m/z 616.2) and isoorientin (m/z 469.2). Chromatographic separation revealed two dominant UV absorption peaks (λ = 254 nm) indicative of distinct chemical classes (Figure 3A). MS/MS fragmentation at 0.068 min (Figure 3B) demonstrated characteristic adducts at m/z 227.1, 255.1 and 283.5, forming a homologous series with 28.0 Da mass differences corresponding to methylene group increments (C_2_H_4_), consistent with reported fragmentation patterns (Ilona et al., 2023). Subsequent analysis at 0.156 min (Figure 3C) identified major ions at m/z 391.2 ([M + K]^+^) and 469.2 ([M + Na]^+^). Molecular mass alignment confirmed target metabolites: gentisin (C_15_H_10_O_5_, MW 258.2), gentiopicroside (C_16_H_20_O_9_, MW 356.3), and isoorientin (C_21_H_20_O_11_, MW 448.4). Based on chromatographic behavior (C18 reverse-phase) and polarity gradients, GSE50 was characterized as predominantly containing gentisin (27.6%), gentiopicroside (7.3%), and isoorientin (65.1%).

### 3.4 Gentisin and isoorientin in GSE and its various fraction solutions may play a greater role than gentiopicroside in promoting fracture repair

**FIGURE 4:**
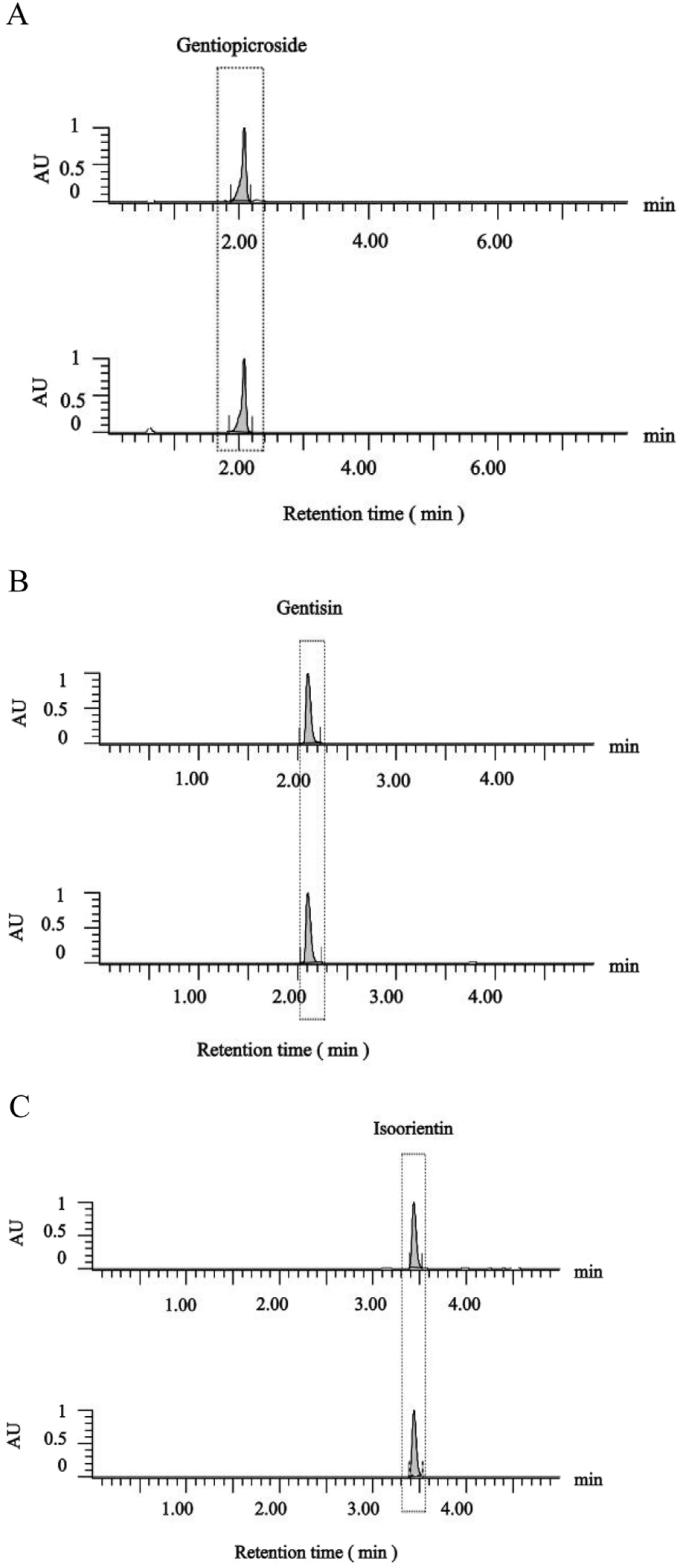
HPLC chromatograms of gentiopicroside **(A)**, gentisin **(B)**, isoorientin **(C)** content in GSE mother liquid and reference standard.

Given the pharmacological significance of active metabolites (gentisin, gentiopicroside, and isoorientin) in Gentiana scabra, quantitative HPLC analysis (Figure 4) revealed distinct concentration profiles across GSE fractions. Gentiopicroside levels in the mother liquor (53.10 mg/g) exceeded pharmacopeial standards (>15.00 mg/g), with further increases observed in GSE70 (84.00 mg/g) and GSE90 (119.39 mg/g). Paradoxically, GSE30 and GSE50 exhibited negligible gentiopicroside content (below detection limits). Histological assessments identified GSE50 and GSE70 as the most potent osteogenic fractions, demonstrating that gentiopicroside exhibited a weaker effect in promoting fracture repair compared to the other two active metabolites (Table 1). Concurrently, a dose-response relationship was observed between the combined content of gentisin and isoorientin in GSE fractions and their osteogenic capacity. Notably, the isoorientin-to-gentisin ratio remained consistent across GSE30, GSE50, and GSE70 (∼2.4). These findings collectively implicate gentisin and isoorientin as key mediators of the therapeutic effects.

**TABLE 1:** The contents of gentiopicroside, gentisin, isoorientinin GSE mother liquid and its various fractions.

GSE fraction	Content of gentisin (mg/g dr. wt)	Content of isoorientinin (mg/g dr. wt)	Content of gentiopicroside (mg/g dr. wt)
	HPLC	HPLC	HPLC
Mother Liquor (ML)	2.13 ± 0.55	11.88 ± 0.27	53.10 ± 0.15
30%	2.93 ± 0.10	7.31 ± 0.13	0.59 ± 0.06
50%	4.28 ± 0.26	10.11 ± 0.27	1.13 ± 0.09
70%	8.01 ± 0.18	19.39 ± 0.69	84.00 ± 0.17
90%	22.68 ± 0.72	34.15 ± 2.06	119.39 ± 0.21

The data were expressed as mean ± SD (n = 3 for HPLC). The purity of gentisin, isoorientinin and gentiopicroside standard used in this study was 99% by HPLC.

## 4 Discussion

The Gentiana scabra extract (GSE) comprises a complex phytochemical profile with demonstrated therapeutic potential in fracture repair. Through chromatographic fractionation, we systematically investigated the osteogenic efficacy of GSE derivatives. Fraction GSE50 exhibited superior fracture healing capacity, as quantitatively validated by radiographic indices (J-image analysis). Phytochemical characterization identified three dominant metabolites in GSE50: gentisin (27.6%), gentiopicroside (7.3%), and isoorientin (65.1%). Intriguingly, pharmacological evaluation revealed an inverse correlation between gentiopicroside content and therapeutic outcomes. Furthermore, a dose-response relationship between gentisin/isoorientin concentrations and bone repair efficacy was observed. In line with previous work (Cao et al., 2011), we found that GSE and its fractions facilitate metatarsal bone fracture healing in chicken model.

Fracture healing is a complex process involving multiple stages, including inflammatory response, angiogenesis, callus formation, and bone remodeling (Liu et al., 2025). This study observed a larger callus volume and higher bone healing scores in GSE and its fractions group, indicating an enhanced fracture healing process. We speculate that this positive therapeutic effect may involve relevant molecular mechanisms. Studies had found that *Gentiana rigescens* inhibits epithelial-mesenchymal transition by regulating the TGF-β1/Smad signaling pathway, downregulates NF-κB protein expression, and alleviates tissue inflammation and collagen deposition (Pi et al., 2021). Other traditional Chinese medicines that promote fracture healing have also been shown to modulate the synthesis and secretion of TGF-β (Ding et al., 2023; Li et al., 2024; Xiong et al., 2025). Research demonstrates that gentiopicroside can reduce the levels of pro-inflammatory cytokines such as tumor necrosis factor-α (TNF-α), interleukin-1β (IL-1β), and interleukin-6 (IL-6) by inhibiting inflammatory signaling pathways like NF-κB (Cao et al., 2025; Liu et al., 2025). This helps control excessive inflammation at the fracture site, creating a favorable environment for healing. Gentiopicroside also alleviates persistent pain by downregulating the protein expression of NR2B receptors in the anterior cingulate cortex (Tian et al., 2024). Isoorientin, as a flavonoid, exerts antioxidant effects and may indirectly promote osteoblast activity (Kuriya et al., 2019; Hu et al., 2021). Gentisin, a natural xanthone compound, primarily functions by inhibiting vascular smooth muscle cell proliferation, as well as exhibiting anti-inflammatory, antioxidant, and antibacterial effects (Sertaç et al., 2025). However, these mechanistic speculations will require future validation through direct molecular biology experiments (such as Western blot, immunohistochemistry, qPCR, etc.). Nevertheless, these findings help explain why GSE and its different fractions could promote fractured bone repair to varying degrees. A possible explanation for this observation was that the combined action of multiple active metabolites in the fractions may limit the full performance of individual metabolites.

This study not only validated the feasibility of GSE and its fractions in bone fracture repair but also isolated and identified the major active metabolites within the GSE50 fraction. Practically, this technology was expected to expand the application scope of GSE and its derived fractions. Furthermore, the fractionation method we developed provides a viable pathway for large-scale production. Future research should focus on elucidating the regulatory mechanisms and signaling pathways of individual monomeric metabolites, as well as exploring their compatibility with medical-grade hydrogels to facilitate eventual commercialization.

Finally, it is necessary to briefly discuss the selection of the animal model in this study. Although rats and rabbits are commonly used models in fracture research, we intentionally chose chickens for the following reasons: First, the primary goal of this study was to conduct preliminary screening of active metabolites in *Gentiana scabra* Bunge that promote fracture repair. Chickens have a high skeletal metabolic rate, and their fracture healing is faster than that of many mammalian species. This shortens the experimental timeline and allows observation of multiple healing stages within a shorter period. Second, the fracture healing process in chickens produces abundant and easily evaluable callus, which is crucial for our quantitative analysis of the callus-to-fracture ratio. Previous studies have confirmed that the chicken model has yielded significant insights into the regulatory mechanisms of osteoclasts (Usui et al., 2008). Therefore, we believe that the data obtained using the chicken model in this study contribute unique and valuable knowledge to the field of fracture healing.

This study had several limitations. First, as noted by the reviewer, a vehicle control group receiving an equivalent volume of 50% ethanol (used as the drug solvent) was not included in the experimental design. Although we observed significant improvements in the treatment group compared to the untreated model control group, we cannot completely rule out potential subtle effects of 50% ethanol itself on local tissue inflammation, cellular activity, or early angiogenesis. Therefore, future studies should incorporate rigorous vehicle controls to definitively attribute the observed therapeutic effects to GSE itself and to provide a purer interpretation of the drug’s mechanism of action.

## Data Availability

The datasets presented in this study can be found in online repositories. The names of the repository/repositories and accession number(s) can be found in the article/supplementary material.

## Abbreviations

GSE, Gentiana scabra alcohol-water extract; GSE and its fraction, GSE mother liquid and different concentrations of ethanol elution solution after passing GSE through D101 resin; HPLC, High-Performance Liquid Chromatography; LC-MS, Liquid Chromatography-Mass Spectrometry.
